# RNA sequencing: from tag-based profiling to resolving complete transcript structure

**DOI:** 10.1007/s00018-014-1637-9

**Published:** 2014-05-15

**Authors:** Eleonora de Klerk, Johan T. den Dunnen, Peter A. C. ‘t Hoen

**Affiliations:** Department of Human Genetics, Leiden University Medical Center, 2300 RC Leiden, The Netherlands

**Keywords:** RNA sequencing, Transcriptome, Gene expression, Transcription regulation, RNA processing, Translation regulation, Methods

## Abstract

Technological advances in the sequencing field support in-depth characterization of the transcriptome. Here, we review genome-wide RNA sequencing methods used to investigate specific aspects of gene expression and its regulation, from transcription to RNA processing and translation. We discuss tag-based methods for studying transcription, alternative initiation and polyadenylation events, shotgun methods for detection of alternative splicing, full-length RNA sequencing for the determination of complete transcript structures, and targeted methods for studying the process of transcription and translation. With the ensemble of technologies available, it is now possible to obtain a comprehensive view on transcriptome complexity and the regulation of transcript diversity.

## Introduction

The transcriptome can be described as the complete collection of RNA molecules expressed in a specific cell type or tissue at a given time. It includes coding RNAs (mRNA) and a multitude of non-coding RNAs (of which ribosomal RNA, transfer RNA, small nuclear RNA, small nucleolar RNA, microRNA, Piwi-interacting RNA, and long non-coding RNA are best characterized). RNA plays a central role in cell biology, where it not only serves as template for protein synthesis but also acts as a structural scaffold and as regulatory molecule during post-transcriptional control of gene expression [1, 2].

The complexity of the transcriptome arises from different layers of regulation of gene expression, including regulation at transcriptional, post-transcriptional and translational level. At the level of transcription initiation, a gene may give rise to multiple transcripts with different transcription start sites and/or first exons [3]. Primary transcripts may undergo alternative splicing and/or alternative polyadenylation, two common RNA processing events which highly increase the number of transcript variants originating from a single gene [4–6]. Eventually, each processed transcript can potentially code for multiple protein products through the use of alternative translation start sites [7, 8]. The effective expression level of a certain gene is also regulated through RNA stability. A gene may be transcribed into (1) an alternatively spliced variant targeted for nonsense-mediated decay [9], or (2) an alternatively polyadenylated variant which has gained or lost regulatory sequences recognized by stabilizing RNA-binding proteins or microRNAs [10–13]. Another form of negative regulation can be observed during translation of a transcript, when protein synthesis is inhibited or reduced due to the use of upstream open reading frames [14, 15].

This review will discuss the major RNA profiling methods (tag-based, shotgun, full-length and targeted), mainly focusing on the class of mRNAs. For each method, we will address its utility for the study of specific RNA transcript regulation and processing events. For technical differences between the mentioned sequencing platforms and extensive descriptions of all the regulatory mechanisms touched in this review we refer to previously published reviews [3–5, 7, 10, 16–20].

### Sequencing platforms

Numerous next-generation sequencing (NGS)-based RNA profiling methods are nowadays available to specifically investigate different levels of regulation.

RNA sequencing methods have been adapted for the most common DNA sequencing platforms [HiSeq systems (Illumina), 454 Genome Sequencer FLX System [Roche], Applied Biosystems SOLiD (Life Technologies), IonTorrent (Life Technologies)]. These platforms require initial reverse transcription of RNA into cDNA. Conversely, the single molecule sequencer HeliScope (Helicos BioSciences) is able to use RNA as a template for sequencing [21, 22] and a few studies have shown its potential [23–26]. A proof of principle for direct RNA sequencing on the PacBio RS platform has also been demonstrated (Pacific Bioscience). However, direct RNA sequencing technologies are currently not available to regular customers.

The sequencing platforms differ also in the number of reads generated, leading to a difference in sensitivity. While common short-read platforms can generate millions of reads (http://res.illumina.com/documents/products/appnotes/appnote_hiseq2500.pdf), allowing an accurate quantitative analysis of high and low abundant transcripts, PacBio currently yields ~50,000 long reads (http://files.pacb.com/pdf/PacBio_RS_II_Brochure.pdf), restricting the number of transcripts that can be detected, unless multiple runs are performed [27–29].

### Overview of RNA sequencing methods

**Fig. 1:**
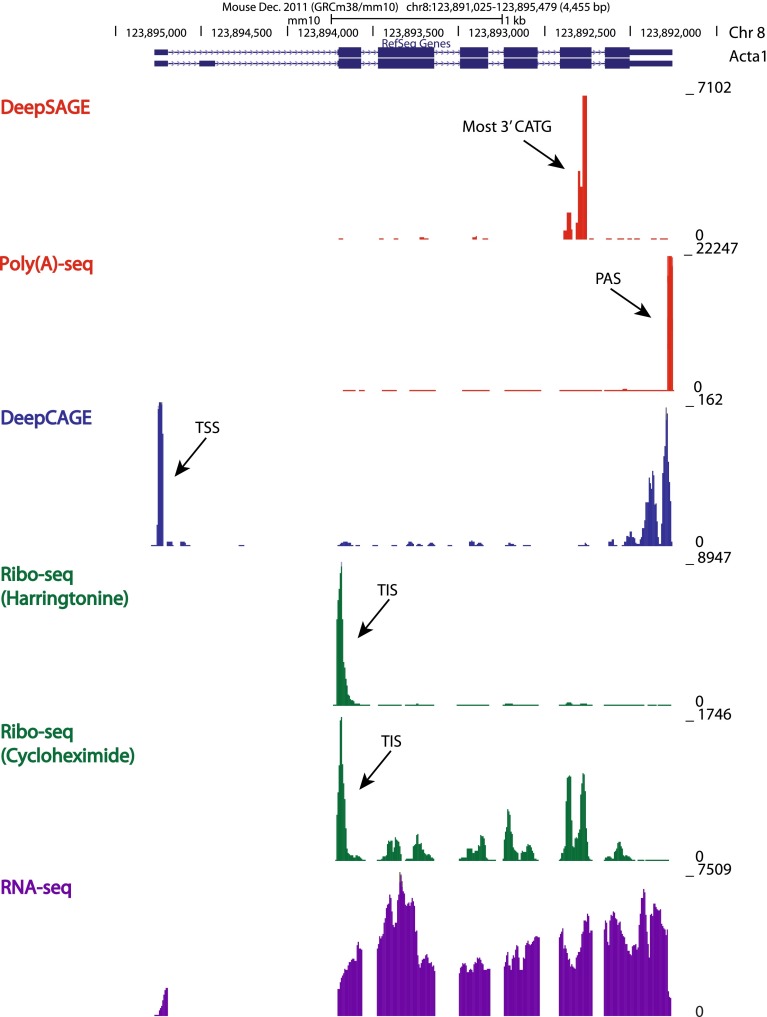
A screenshot from UCSC Genome Browser (http://genome.ucsc.edu) displaying the different regions sequenced by tag-based and shotgun methods in Acta1 gene. The *y*-axis represents the coverage, corresponding to the number of reads mapping at each location. Six independent traces are shown. The top two traces (in *red*) show a peak at the most 3′ CATG site and at the exact polyadenylation site (PAS, indicated by an *arrow*) detected by DeepSAGE and Poly(A)-seq, respectively. The third trace (in *blue*) shows a peak at the transcription start site (TSS, indicated by an *arrow*) detected by DeepCAGE. The fourth trace (in *green*) shows a peak at the translation start site (TIS, indicated by an *arrow*) detected by ribosome profiling based on harringtonine treatment. The fifth trace (also in *green*) shows a major peak at the detected translation start site (TIS, indicated by *arrow*) and a lower coverage at each translated exons, detected by ribosome profiling based on cycloheximide treatment. The last trace (in *purple*) shows a typical RNA-seq profile, where all exons and untranslated regions are detected. On top of the coverage tracks, the RefSeq gene track shows two transcript variants for Acta1, with exons shown as *thick boxes*, untranslated regions as *thin boxes* and introns as *consecutive arrows*

**Fig. 2:**
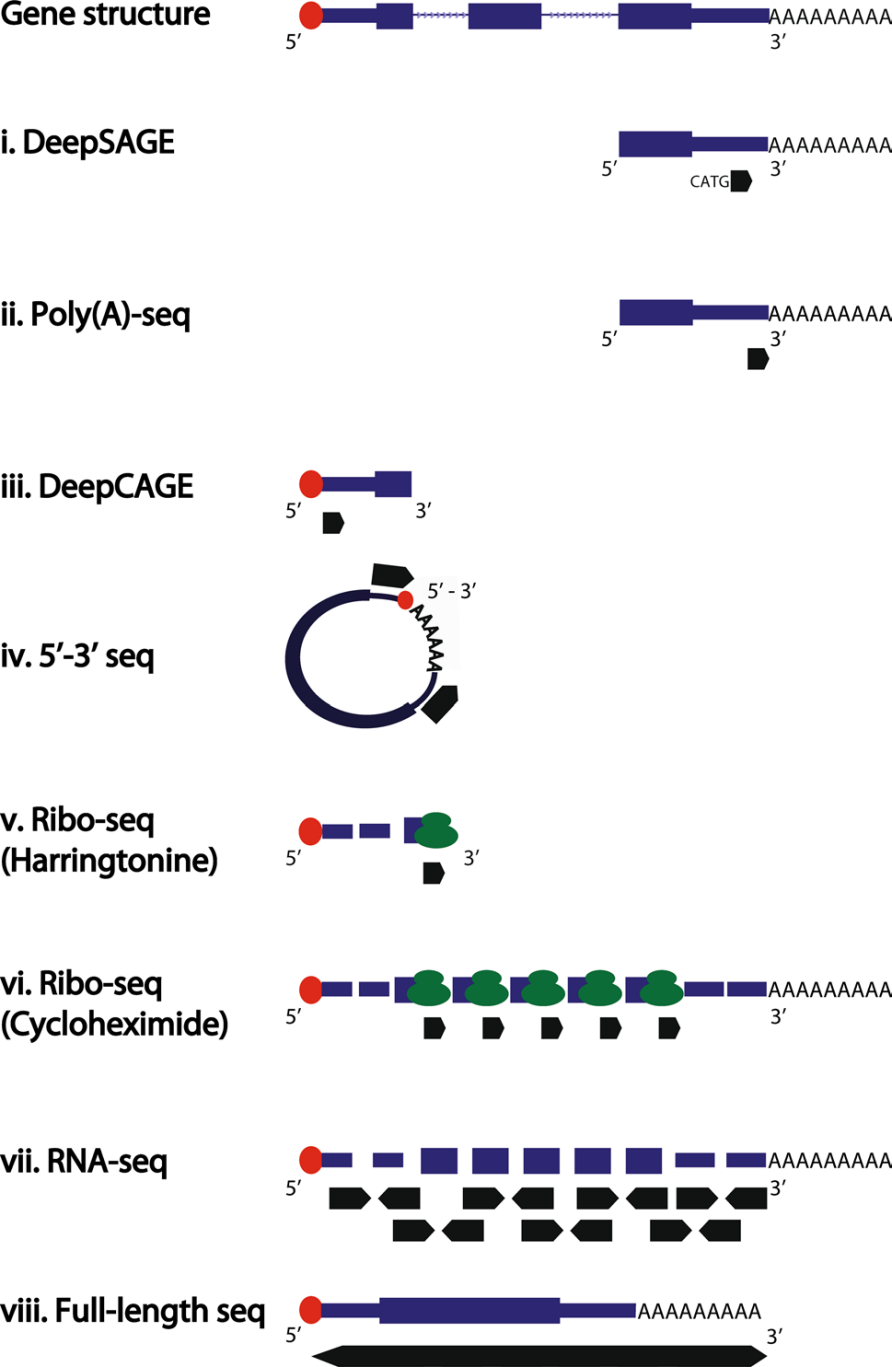
Schematic representation of sequencing reads generated by tag-based (*i*–*iv*), shotgun (*v*–*vii*) or full-length (*viii*) sequencing. *Thick black arrows* indicate the sequenced reads. Paired-end reads are displayed by *two opposite black arrows*. *Red circles* indicate the 5′ cap structure. Ribosomes are displayed in *green*. The complete gene model is displayed on *top*, with exons shown as *thick boxes*, untranslated regions as *thin boxes* and introns as consecutive *thin arrows*

Whereas some RNA sequencing methods focus on a particular region of the transcript and are zooming in on specific RNA processing events, others provide a more comprehensive picture of the transcript, simultaneously characterizing different processing events (Fig. 1). In this perspective, we can classify RNA sequencing methods into two categories: (1) tag-based methods, where only a short fragment (tag) at a defined position in each RNA molecule is sequenced, and (2) shotgun methods, where the molecule is divided and sequenced in multiple fragments and reconstruction of the original transcript is attempted through computational and statistical approaches (Fig. 2). A completely different categorization is needed for RNA sequencing methods based on the PacBio sequencing platform. PacBio long-read sequencing provides full-length transcript sequencing, allowing an exact characterization of the structure of the transcript [28, 30]. In this way, different RNA processing events can be simultaneously detected and specifically assigned to a certain transcript, without the ambiguity faced in all other shotgun methods developed for short-read sequencing platforms.

It is important to note that each of these methods capture RNA molecules in different ways, some rely on the presence of the 5′-cap or the poly(A) tail, others allow a full sampling of the transcriptome by capturing also non-capped and non-polyadenylated molecules. The transcripts detected by different techniques are therefore only partially overlapping. Another issue to consider is the transcript’s orientation. While all tag-based methods are strand specific, meaning that they preserve information about the transcript’s orientation, shotgun methods may be strand specific or not strand specific. Strand specificity is important to determine the exact gene expression levels in the presence of antisense transcription.

These advanced RNA sequencing methods and platforms generate a huge amount of data, up to millions of reads, giving us the possibility to understand the complexity of the transcriptome and its fine regulation. To correctly interpret sequencing data and reach a full understanding of the hidden biological meaning in it, a parallel development of statistical and computational approaches is fundamental. Numerous algorithms have been developed to detect differentially expressed genes and spliced variants. For an extensive comparison of some of the most commonly used methods, and for a general overview of the computational challenges, we refer to [29, 31, 32]. Moreover, dedicated algorithms to identify switches between polyadenylation [33, 34] or transcription start sites [35, 36] have been developed.

## Tag-based methods

In tag-based methods, each transcript is represented by a unique tag. Initially, tag-based approaches were developed as a sequence-based method to measure transcript abundance and identify differentially expressed genes, assuming that the number of tags (counts) directly corresponds to the abundance of the mRNA molecules. The reduced complexity of the sample, obtained by sequencing a defined region, was essential to make the Sanger-based methods affordable. When NGS technology became available, the high number of reads that could be generated facilitated differential gene expression analysis. A transcript length bias in the quantification of gene expression levels, such as observed for shotgun methods [37, 38], is not encountered in tag-based methods. This makes tag-based method a potentially less biased approach when studying gene expression. Moreover, all tag-based methods are by definition strand specific.

Recently, an increased interest in the determination of transcripts’ structure led to the development of numerous directed tag-based strategies which aim to precisely define 3′ and 5′ transcript ends. We will refer to them as 3′ end sequencing and 5′ end sequencing methods.

### 3′ end sequencing

3′ end sequencing methods specifically focus on the end of the transcript, allowing the detection of transcripts which differ in the 3′-terminal exon used or in the length of their 3′ untranslated region (3′-UTR). Different 3′ ends arise from alternative polyadenylation of pre-mRNAs [39–41]. Alternative polyadenylation is a common regulatory mechanism [42–46] and represents an important layer of regulation of gene expression at post-transcriptional level.

The complexity of the transcriptome highly increases through the use of alternative polyadenylation sites within different exons/introns or within the same 3′-UTR, the first giving rise to transcript variants coding for different protein isoforms and the second giving rise to transcript variants potentially differing in stability [42, 44, 45, 47–49].

A variety of 3′ end sequencing methods have been developed in the last years, from serial analysis of gene expression (SAGE)-like methods to more dedicated protocols, where the detection of the actual polyadenylation site used is even more precise. We review some of these methods, and assess the level of precision in which polyadenylation sites are determined.

**Fig. 3:**
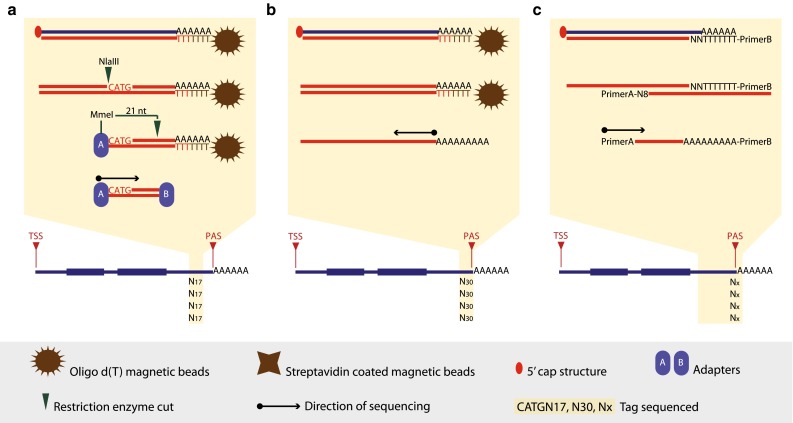
3′ end sequencing methods. **a** In DeepSAGE [50] poly(A)^+^ RNAs are captured by oligo d(T) magnetic beads and reverse transcribed. cDNA is digested with NlaIII and adapter A is ligated. A second digestion with MmeI generates a 21-bp tag, and adaptor B is ligated to the 3′ end. The construct is amplified and sequenced from adapter A. **b** In HeliScope-based Poly(A)seq [33] poly(A)^+^ RNAs are captured by oligo d(T) magnetic beads and reverse transcribed. Second-strand cDNA molecules are hybridized to the Helicos flow cell and sequenced starting precisely at the polyadenylation site. **c** In MAPS [61] first- and second-strand syntheses are carried out using oligo d(T) linked to primer B and random primers linked to primer, respectively. The construct is amplified and sequenced starting from the 5′ end of the construct

DeepSAGE [50] represents the first high-throughput tag-based method developed to generate tags at the most 3′ end of a transcript. DGE [51], Tag-Seq [52] and HT-SuperSAGE [53] are improved versions which have been adapted to different sequencing platforms. All these approaches are based on the SAGE method described by Velculescu et al. [54]. Minor differences characterize these techniques, such as the length of the tag (21 or 25–26 nt), the restriction enzymes used to release the 3′ end of a transcript and generate a unique tag (NlaIII/MmeI or NlaIII/EcoP15I), and the sequencing platform used. Except for these minor differences, the steps necessary to generate a sequencing library are similar (Fig. 3a).The first steps consist in capturing all polyadenylated transcripts and converting the RNA molecules into double-stranded cDNA molecules. The cDNA molecules are then cut at the most 3′ CATG by enzymatic digestion and ligated to a 5′ adapter, which introduces a recognition site for a specific restriction enzyme (MmeI/EcoP15I). A second digestion, downstream of the incorporated restriction site, produces a short fragment (tag of 21 or 25–26 nt) which is then ligated to a 3′ adapter. Both adapters make the cDNA tag suitable for amplification and high-throughput sequencing.

Different studies have shown that SAGE-like methods are suitable to detect alternative polyadenylation events [42, 51, 55–57]. Nonetheless, the possibility to distinguish transcripts with different 3′ end relies on the presence of a restriction site in the sequence between the two alternative polyadenylation sites. All transcripts with alternative 3′ ends lacking restriction sites in between the polyadenylation sites are, therefore, missed. The same applies for transcripts which do not contain that specific restriction site. According to RefSeq human transcript database, ~1 % of the transcripts lack an NlaIII recognition site, meaning that almost 1000 transcripts are not accessible to SAGE-like approaches [58]. Another limitation of these methods is that they do not give information regarding the position of the polyadenylation site.

To overcome the limitations observed in all SAGE-like methods, several dedicated protocols have been developed to specifically characterize polyadenylation sites and quantify their relative usage genome wide [21, 22, 33, 46, 48, 59–70] (Fig. 3b, c). These methods do not rely on the presence of a specific restriction enzyme site and therefore detect all polyadenylation sites.

Limitations in the detection of the exact polyadenylation site location and biased quantifications may arise due to various steps involved in the preparation of the sequencing library. Oligo(dT) priming, DNA or RNA ligase-mediated adapter ligation, reverse transcription and amplification represent the main sources of bias.

The available poly(A) site sequencing protocols may differ in the level of precision in which the polyadenylation site is determined, in the number of possible biasing steps introduced and in the number of false polyadenylation sites detected, mainly arising from internal priming events.

The main technical differences between the reviewed methods are summarized in Table 1.

**Table 1:** Polyadenylation site (PAS) sequencing protocols

	PAS-Seq	SAPAS	PolyA-seq	A-seq	MAPS	3′Seq	3P-Seq	3′READS	3′T-fill	de Klerk et al.	Ozsolak et al.
Reverse transcription	▲	▲	▲	▲	▲	▲	▲	▲	▲	▲	
Oligo(dT)-based	▲	▲	▲	▲	▲	▲			▲		
DNA ligase-mediated adapter ligation						▲			▲		
RNA ligase-mediated adapter ligation				▲			▲	▲			
Sequencing starts next or at PAS	▲ (*)		▲ (*)	▲ (*)			▲ (*)	▲ (*)	▲ (**)	▲ (**)	▲ (**)
Sequencing starts at poly(A) tail		▲									
Sequencing starts at 5′ end					▲	▲					

* Sequencing starts next to PAS

** Sequencing starts at exact PAS

Internal priming events remain one of the limitations of all methods based on oligo(dT) priming [46, 60–62, 66, 69, 71]. Internal priming can occur due to priming of oligo(dT) on internal A-rich regions of the transcript, yielding artifacts which are difficult to distinguish from authentic polyadenylation sites.

Different approaches have been taken to minimize internal priming artifacts. In 3P-Seq [63], ligation of a biotinylated double-stranded oligo (containing an overhanging stretch of Ts) to the end of the poly(A) tail is used to eliminate the chance of priming in internal poly(A) stretches. In another method, 3′READS [48], discrimination of 3′ poly(A) tails from internal A-rich sequences is achieved by capturing fragmented RNA onto beads coated with a chimeric oligonucleotide consisting of thymidines (Ts) at the 5′ and uridines (Us) at the 3′ end (CU5T45). Subsequently, RNaseH digestion is used to release the molecules from the beads and to remove most of the As of the poly(A) tail. This method enriches for RNAs with longer A stretches.

Wang et al. [68] used a computational analysis to distinguish authentic polyadenylation sites from potential internal priming events based on the distinct pattern of nucleotide composition of the 3′ end region. This method is compatible with any 3′ end sequencing technology.

Next to differences in dealing with the internal priming issue, protocols display different degrees of resolution in the identification of the exact polyadenylation sites. If sequencing starts from the 5′ end of the library construct [59, 61, 64, 71], there is a chance that a fraction of reads will not reach the polyadenylation site. If sequencing starts at the very 3′ end of the library construct [62], including the stretch of As, other issues may arise, such as polymerase slippage or mispriming of the sequencing oligo, due to the presence of the homopolymeric stretch. The 3P-Seq approach described above [63] overcomes this last issue by digesting the poly(A) tail before incorporating the adapters necessary for amplification and sequencing. The PAS-Seq [46] approach avoids sequencing the poly(A) tail using a sequencing primer with an oligo(dT) extension at the 3′ end.

Another method which avoids sequencing through the poly(A) tail is described by Wilkening et al. [69]. In this method, named 3′T-fill, the poly(A) stretch is filled in with dTTPs before the sequencing reaction starts.

A more direct approach is described by de Klerk et al. [33]. Their method, based on the HeliScope single molecule sequencer technology, allows to start sequencing directly after the 5′ end of the poly(A) tail, thus at the exact polyadenylation site. Molecules are directly hybridized, through their poly(A) tail, to a flow cell containing oligo(dT) probes. The poly(A) stretch downstream of each polyadenylation site makes the second-strand cDNA molecules directly amenable for sequencing, with the advantage that the first nucleotide on the 5′ end of each sequenced molecule represents the poly(A) addition site.

An even less biased approach is described by Ozsolak et al. [21, 22], and is based on direct RNA sequencing (DRS). All poly(A)-containing RNAs are sequenced starting from the polyadenylation site, without reverse transcription, right after one single enzymatic reaction consisting in the addition of dideoxy terminators at the end of the poly(A) tail. This is done to prevent extension at the 3′ end of mRNAs which are not perfectly hybridized to the poly(T) stretch of the flow cell surface.

Accurate detection of polyadenylation sites can also be achieved on the PacBio-RS single molecule sequencing platform. Here, transcripts are converted into a circular double-stranded DNA template capped by hairpin loops at both 3′ and 5′ ends [72]. Since the full-length cDNA molecule is incorporated in a circular template, the poly(A) tail will be present, allowing the detection of the exact position of the polyadenylation site and the length on the poly(A) tail.

Methods relying on enzymatic ligation of adapter sequences to RNA molecules (such as A-Seq [66], 3P-Seq [63] and 3′READS [48]), are known to be non-random, compromising quantification [73, 74]. Ligation steps may be avoided using the template switch reverse transcription approach. Methods such as PAS-Seq [46], SAPAS [62] and PolyA-seq [60], use this approach to incorporate known sequences at both ends of cDNA molecules during first-strand synthesis. Despite this, other artifacts may be introduced, e.g., through a process called strand invasion [75].

### 5′ end sequencing

5′ end sequencing methods can be considered as a mirror approach of the 3′ end sequencing methods, as they generate tags at the 5′ end of a transcript. 5′ end sequencing methods have been developed to specifically identify transcription start sites (TSS) and (proximal) promoters. The knowledge of the exact position of a transcription start site can also be used to investigate promoter usage and to identify transcription factor binding sites in these promoters [76].

The detection of the exact transcription start sites is highly important since alternative transcription start sites can lead to the formation of protein isoforms with totally different biological functions. Alternatively, shorter or longer 5′-UTRs may influence the efficiency of protein translation [14, 15].

The number of 5′ end sequencing methods available is restricted compared to the number of 3′ end sequencing approaches. A possible reason might be that the first method published, named DeepCAGE [77–79], already efficiently detected 5′ ends of transcripts, with a high level of precision.

Whereas SAGE-like methods are restricted to the use of restriction enzymes and therefore to the presence and location of restriction sites, CAGE-like methods are based on the 5′ cap structure of a transcript, and can theoretically detect all capped 5′ ends of mRNA molecules. On the other hand, these methods are not suitable for non-capped transcripts.

DeepCAGE represents an improved NGS version of the previously published CAGE protocols [80, 81]. This technique makes use of the cap trapper method [82] to capture the 5′-cap structure of RNA molecules. Trapped RNAs are converted to cDNAs, and an adapter is ligated to the 3′ end of the cDNAs. The adapter is used to introduce a recognition site for a specific restriction enzyme (Mme1 or EcoP15I), which is able to cut 21 or 25–27 nt downstream, generating the tag desired. After synthesis of the second cDNA strand, the double-stranded cDNA fragment is ligated to a second adapter, necessary for amplification before sequencing.

DeepCAGE libraries have been analyzed on common DNA-based sequencing platforms (Illumina, 454) but also on the Helicos single molecule sequencer [83, 84]. The Helicos-based DeepCAGE method (called Heliscope-CAGE) is a simplified method which consists of only three main steps: first-strand cDNA synthesis, 5′-cap trapping and poly(A) tailing of the 3′ ends. Heliscope-CAGE has the advantage to avoid second-strand synthesis, amplification, ligation, and digestion, reducing possible quantification bias that might arise from each of these steps. Molecules can be hybridized to the flow cell and sequencing can start directly after filling up the poly(A) tail.

Both DeepCAGE and HeliscopeCAGE are based on the cap-trapper method. A different approach is described by Salimullah et al. [85] in their protocol named NanoCAGE, initially developed by Plessy et al. [86]. NanoCAGE uses the template-switching method for reverse transcription. Compared to cap-trapper-based methods, an advantage of this approach is the low amount of starting material (~50 ng instead of ~5 µg) required and the possibility to sequence not only a single tag at the transcription start site, but also a second tag in a downstream exon. The position of the second tag is random, since it depends on the position of the random primer used during second-strand synthesis. Paired-end sequencing of NanoCAGE libraries will therefore provide extra information on the structure of the transcript compared to DeepCAGE methods. The same approach is used in the method called CAGEscan [86]. The limitation of NanoCAGE and CAGEscan lies in the possible artifacts introduced by template switching [75].

All CAGE-like methods discussed so far are limited in their ability to correctly detect alternative transcription start sites, due to a phenomenon called ‘exon painting’ [55, 83, 87]. The term ‘exon painting’ is used to indicate the presence of multiple CAGE peaks in exonic regions, next to the expected CAGE peak at the 5′ end of the transcript. This phenomenon is not caused by a technical artifact, but more likely arises from recapping of processed transcripts [87]. To limit the number of false alternative transcription start sites detected, only TSS in intergenic regions are considered [55].

### 5′ and 3′ end sequencing

The detection of alternative transcription start sites and alternative polyadenylation sites by tag-based methods, which focus on the 5′ and 3′ end of a transcript, respectively, is a proven method to characterize transcript structure. Nevertheless, the full information about transcript structure is missing. To overcome this limitation, tag-based methods able to detect the co-occurrence of a specific transcription start site and a polyadenylation site has been developed. Methods able to determine both ends are called RNA-PET [88] and TIF-Seq [89]. RNA-PET is a paired-end tag approach, where detection of both 3′ and 5′ ends occurs through paired-end sequencing. The initial step consists of capturing the 5′-cap structure by cap-trapper and synthesizing full-length cDNA. The double-stranded cDNA molecules are ligated to specific adapters which allow the formation of a circular template and the introduction of two restriction sites for EcoP15I. The restriction sites are inversely oriented, allowing the double cleavage of the PET construct, yielding a fragment of 27 nt from both the 3′ and the 5′ ends.

In TIF-Seq full mRNAs are first ligated to a single-strand oligo by oligo-capping. Then mRNAs are converted to cDNAs by reverse transcription and amplified using biotinylated primers. The double-stranded cDNA molecules are circularized through an intramolecular ligation, and fragmented by sonication. Fragments containing both 3′ and 5′ ends are captured by streptavidin-coated beads and ligated to adapters for amplification and paired-end sequencing. An advantage of both paired-end tag approaches is the ability to detect fusion transcripts. On the other hand, generation of full-length cDNAs from long transcripts still represents a technical limitation for any 5′3-sequencing method.

## Shotgun methods

The advantage of a shotgun, sequence-it-all method, over a tag-based method, is the ability to quantify the expression level of each exon within a transcript, estimate their percent inclusion level and detect (differential) alternative splicing events. However, it is difficult to identify the exact 3′ and 5′ ends of transcripts due to various technical biases (such as random hexamer priming or oligo dT priming) leading to underrepresentation of sequences near 5′ and 3′ ends [90, 91].

The term RNA-seq is used to indicate any RNA sequencing method based on a shotgun approach. Numerous protocols have been published so far, but they have many steps in common: fragmentation (which can occur at RNA level or cDNA level, where RNA fragmentation appears to introduce less bias [92]), conversion of the RNA into cDNA (performed by oligo dT or random primers), second-strand synthesis, ligation of adapter sequences at the 3′ and 5′ ends (at RNA or DNA level) and final amplification. RNA-seq can focus only on polyadenylated RNA molecules (mainly mRNAs but also some lncRNAs, snoRNAs, pseudogenes and histones [93–95]) if poly(A)^+^ RNAs are selected prior to fragmentation, or may also include non-polyadenylated RNAs if no selection is performed. In the latter case, ribosomal RNA (more than 80 % of the total RNA pool [96]) needs to be depleted prior to fragmentation. It is, therefore, clear that differences in capturing of the mRNA part of the transcriptome lead to a partial overlap in the type of detected transcripts. Moreover, different protocols may affect the abundance and the distribution of the sequenced reads [97]. This makes it difficult to compare results from experiments with different library preparation protocols.

Whereas all tag-based methods are by definition strand specific, the first RNA-seq methods were not strand specific [92], as the orientation of the molecule was lost during random-primed cDNA synthesis. In the last years, numerous strand-specific RNA-seq protocols have been developed (Table 2) [98–102]. Maintaining strand information is important given the widespread occurrence of antisense transcripts with a, likely regulatory, biological function.

**Table 2:** RNA-seq protocols

	Mortazavi et al.	Lister et al.	He et al.	Parkhomchuk et al.
RNA fragmentation	▲	▲		
cDNA fragmentation			▲	▲
RNA ligase-mediated adapter ligation		▲		
Random hexamers priming	▲		▲	▲
Oligo(dT) priming				▲
Adapter priming		▲		
Bisulfite treatment			▲	
Deoxy-UTP incorporation in dsDNA				▲
Strand specific		▲	▲	▲

Strand-specific methods can be classified into two categories: (1) RNA-seq methods based on ligation of two different adaptors in a known orientation relative to the 5′ and 3′ ends, and (2) RNA-seq methods based on chemical modification of the RNA, either by bisulfite treatment or by the incorporation of dUTPs during the second-strand cDNA synthesis. In both cases, the non-modified strand is degraded enzymatically. According to a comparative study published by Levin et al. [103], where 13 different protocols have been analyzed based on their strand specificity, the coverage along all exons and the accuracy in quantification, the dUTP approach was the best performing protocol. Nevertheless, in all strand-specific RNA-seq protocols a fraction of antisense reads will be generated, for example when RNA molecules fold back on themselves. Depending on the protocol, the percentage of antisense reads from sense transcripts amounts to 1–12 % [103]. Therefore, additional analytical approaches are required to discriminate naturally occurring antisense transcripts from artifacts.

Shotgun sequencing methods have the potential to identify alternative splicing events. Algorithms deriving transcript structure from short reads mostly use a combination of coverage patterns and exon–exon spanning reads, and read pair information. To be able to detect alternative spliced variants, a certain coverage is necessary. Therefore, low expressed genes will give less information than highly expressed genes, unless a large number of reads are generated. A discussion of these algorithms falls outside the scope of this review. The reader is referred to [29, 104].

## Full-length sequencing

One of the main limitations of all short-read shotgun methods is the inability to directly characterize the structure of a transcript and/or to discriminate different alleles. Additional computational and statistical approaches are required to reconstruct the transcript, and the short fragment sizes limit the reconstruction to local regions of the transcripts.

The PacBio system is the only available platform potentially able to produce reads with a length up to ~30 kb. However, the limitation faced at the moment is the production of full-length double-stranded cDNAs [28].

Different approaches are used to create full-length cDNAs suitable for full-length transcript sequencing. One of the possible approaches is based on template switching, consisting in the addition of a non-templated poly-cytosine tail to the 3′ end of the first-strand cDNA molecule through the terminal transferase activity of the MMLV reverse transcriptase. The addition of a poly-(C) tail allows the hybridization of an adapter with a poly(G) tail if the first-strand cDNA synthesis has reached the 5′ end of the transcript. A disadvantage of this approach is that degraded mRNAs containing a poly(A) tail will also be converted into cDNAs, simply due to the fact that cDNA synthesis starts at the poly(A) tail. Distinction between full-length transcripts and partially degraded transcripts will therefore be impossible.

A different approach based on the isolation of properly 5′-capped RNA molecules is also extensively used. It is based on first-strand cDNA synthesis starting at the poly(A) tail, followed by digestion of unconverted RNAs and capture of the 5′-cap. Only molecules where the cDNA synthesis has reached the 5′ cap will be used for second-strand synthesis.

Minor improvements in cDNA length have been observed in recent template switch-based methods like Smart-seq2 [105], where the majority of the cDNA molecules reach a read length of 2 kb.

Independently from which approach is used to generate full-length cDNAs, for PacBio sequencing these are converted into a SMRTbell library [72], consisting of double-stranded cDNA molecules capped by two harpin adapters on both side. The hairpin adapters are used to convert the linear double-stranded cDNAs into circular cDNA molecules, which due to this structure and long-read lengths will be sequenced multiple times by the same polymerase. Fragmentation and amplification steps are not performed, with the advantage that any possible technical artifact commonly faced in most of the current methods is avoided.

Taking into account the actual limitations observed in full-length cDNA preparation, full-length sequencing on PacBio still represents a unique approach to interrogate transcript structure on a single molecule level. Unfortunately, the number of reads offered by the PacBio technology is limited, and full characterization of a transcriptome requires performing of many runs [27, 28] and is costly.

## Immunoprecipitation-based methods

Whereas previous methods usually reflect steady-state RNA levels, there are also dedicated methods available to monitor active transcription. A first approach is the immunoprecipitation of genomic DNA bound by RNA Polymerase II [106]. Depending on the antibody used, only transcription initiation complexes are immunoprecipitated or also actively transcribed DNA. Alternatively, nascent RNA molecules can be sequenced by NET-seq [107] (native elongating transcript sequencing). In this approach, the ternary complex formed by the RNA pol II, DNA and RNA is immunoprecipitated. Crosslinking can be avoided due to the stable ternary complex.

RNA immunoprecipitation-based methods are also used to understand how protein–RNA complexes interactions regulate gene expression at transcriptional and post-transcriptional level. Various targeted approaches have been developed to investigate the interaction between RNA-binding proteins and their target RNA molecules (Table 3).

**Table 3:** Immunoprecipitation-based protocols

	NET-seq	HITS-CLIP	CLIP-seq	PAR-CLIP	iCLIP
Crosslink UV 254 nm		▲	▲		▲
Crosslink UV 365 nm				▲	
RNA ligase-mediated adapter ligation	▲	▲	▲	▲	▲
Reverse transcription	▲	▲	▲	▲	▲
Photoreactive ribonucleoside analogs				▲	
Identification of precise crosslinked site				▲	▲

HITS-CLIP [108] and CLIP-seq [109] represent the first high-throughput methods developed to generate genome-wide RNA–protein interaction maps. Both methods are based on the crosslinking-immunopurification (CLIP) strategy [110, 111], which relies on the principle that ultraviolet light causes the formation of a covalent bound between RNAs and proteins in direct contact.

Cells or tissues can be irradiated in vivo, and after cell lysis the crosslinked RNA–protein complexes can be purified by immunoprecipitation using specific antibodies. To be able to map each binding site, RNA is digested up to a length of ~50 nt, reverse transcribed after RNA adapter ligation, and amplified prior sequencing. In the traditional CLIP method the resolution is low, since the mapped binding sites correspond to the total length of the fragmented co-purified RNAs. Another limitation is represented by the low efficiency of crosslinking using UV light at a wavelength of 254 nm. Different approaches, such as PAR-CLIP [112, 113] and iCLIP [114], have been developed to more precisely map the exact binding sites at nucleotide resolution and to increase the efficiency of the crosslinking.

PAR-CLIP [112, 113] (photoactivatable-ribonucleoside-enhanced crosslinking and immunoprecipitation) is based on the incorporation of photoreactive ribonucleoside analogs (4-thiouridine or 6-thioguanosine) into newly synthesized RNAs. The use of ribonucleoside analogs leads to two advantages: they allow crosslinking with UV light at 365 nm (more efficient than the crosslinking at 254 nm), and they lead to a base transition during reverse transcription (thymidine to cytidine or guanosine to adenosine when using 4-thiouridine or 6-thioguanosine, respectively) which can be used to exactly define the crosslink site at nucleotide resolution.

HITS-CLIP, CLIP-seq and PAR-CLIP face the problem of truncated cDNAs generated during reverse transcription. Reverse transcription can stop due to the presence of undigested peptides which are still crosslinked to the RNA molecules. Truncated cDNAs are usually lost because they cannot be amplified, due to the missing 5′ adapter primer.

iCLIP [114] makes use of partial peptide digestion to appositely create truncated cDNA molecules, which can be converted into circular cDNA molecules. The crosslink position can be exactly defined since it corresponds to one nucleotide upstream of the truncation site.

Any of the CLIP methods mentioned above require numerous enzymatic steps which can bias the detection of true binding sites (from RNA and protein digestion, to RNA ligase-mediated adapter ligation, reverse transcription and amplification). Moreover, even though a crosslinking at 365 nm is generally considered more efficient, the efficiency of a crosslink might differ from protein to protein [115].

Most of the CLIP-based studies performed so far focus on splicing factors [108, 109, 114].

## Ribosome profiling

All methods discussed so far focus on measuring the abundance and characterizing the structure of a transcript, or defining its interaction with RNA-binding proteins. The information derived is therefore restricted to the composition of the transcriptome. However, transcript levels are not necessarily a good approximation of protein levels because the process of translation is also highly controlled, probably to the same extent as transcription or splicing [116]. Ribosome-associated mRNA levels are a better proxy for protein levels than total mRNA levels [117].

Ribosome profiling (also called Ribo-seq) [117–119] has been developed to study the process of translation and its efficiency. This method is also often combined with RNA-seq to define untranslated RNAs (e.g., lncRNAs), whether all alternative transcripts are actively translated and to study the extent of regulation at the level of transcription and translation.

Ribosome profiling is a shotgun method based on deep sequencing of ribosome-protected mRNA fragments, which allow to determine which transcript is actively translated at a specific moment in the cell, the rate of translation, the reading frame used and thereby the exact protein product. The technique is based on the observation that ribosomes bound to mRNA molecules protect ~28 nt fragments from nuclease digestion (ribosome footprints). After halting translation, ribosome-bound mRNAs are digested and the ribosome:mRNA complexes (monosomes) are recovered by ultracentrifugation on sucrose gradients or by size-exclusion chromatography. The short protected fragments are released from the monosomes, and converted into a cDNA library, which can be amplified and sequenced. Different variants of the original protocol have been developed to study translational control at different levels. Using drugs arresting ribosome initiation complexes, such as harringtonine or lactimidomycin, it is possible to detect alternative translation start sites or regulatory upstream open reading frames. By inhibiting ribosome translocation with cycloheximide or by thermal freezing, it is possible to quantify the level of translation, to identify the translational reading frame, potential reading frame switches, and to investigate ribosome pausing.

It has been shown that some of the methods commonly used to halt translation may lead to artifacts. Cycloheximide is known to cause a profound accumulation of ribosomes at the translation initiation codon, due do the fact that translation can still initiate while elongation is already blocked [117]. Harringtonine, on the contrary, might fail in halting the ribosomes at the start codon [8]. No disadvantages have been observed so far when halting translation using lactimidomycin, which currently seems to be the method of choice [8].

## From bulk transcriptome to single cell

Large required amounts of input material represent an obstacle when studying rare and heterogeneous cell populations, micro-dissected tissues, subcellular fractions or simply when there is a limited accessible quantity of RNA from patients. Therefore, some RNA profiling methods are limited to bulk transcriptome analysis of large numbers of cells or pieces of tissues.

The targeted approaches, such as the immunoprecipitation-based methods and the ribosome profiling method, require the highest amount of input material, in the range of millions of cells. The suggested amount of RNA for a PAR-CliP experiment ranges between 100 and 400 million cells [113], but iCLIP experiments can be performed in <10 million cells [114], and the same applies for ribosome profiling experiments [119]. None of these approaches has been so far optimized to analyze transcriptome from single cells or from a small population of cells.

PacBio long-read sequencing also requires a high amount of input RNA, in the range of hundreds of thousands of cells. Successful full-length libraries have been generated starting from ~10 µg of total RNA [28] or ~1 µg of poly(A)^+^ RNA [27].

Tag-based and shotgun methods have been extensively improved with regards to the amount of starting material. While the older DeepCAGE approach required ~50 µg of total RNA [79], the single molecule HeliScopeCAGE method requires only ~5 µg of total RNA [83] and the nanoCAGE approach has been optimized to be used with an amount of total RNA ranging from 10 ng to 1 µg (even though the most reliable results are obtained when using at least 50 ng of total RNA) [86]. This allows investigating 5′ ends of transcripts from a small population of cells.

The 3′ end sequencing methods generally require low amounts of input RNA. Even though some poly(A) sequencing methods requires between 10 and 50 µg of total RNA [62, 63, 66] or between 0.5 and 1 µg of poly(A)^+^ RNA [46, 64], others, such as 3Seq [68], the Helicos-based poly(A) seq [33], PolyA-seq [60] and MAPS [61], require only between 0.5 and 3 µg of total RNA. The fact that there are no single-cell studies based on poly(A) sequencing does not imply their unfeasibility, given the fact that the sample preparation for some of these methods partially resemble the one for RNA-seq libraries.

RNA-seq remains at the moment the only method which has been used for whole-transcriptome single-cell sequencing.

One of the main challenges in single-cell RNA-seq is the ability to distinguish between biological variation and technical variation, which suffers from biases introduced during cDNA synthesis and amplification. Next to the ambiguity in the quantification, when the starting amount is lowered to single-cell level, it also becomes difficult to detect lowly expressed transcripts [120]. Recently, numerous RNA-seq methods specific for single-cell transcriptome sequencing have been developed to decrease technical variation [120] [121], together with statistical methods to distinguish the true biological variability [122]. A comparison of commercially available kits showed that single-cell RNA sequencing can detect the same transcriptome complexity observed with standard RNA-seq on millions of cells [123]. The advantage of single-cell RNA sequencing over standard RNA-seq on a bulk of cells relies in the possibility to detect expression differences which could be overlooked when looking at an heterogeneous population of cells, such as allele-specific expression [124]. Even though studies have shown the possibility to detect splicing events [120], alternative 3′ or 5′ ends [125–127], SNPs and mutations [120], in single-cell analysis further improvements are still needed to decrease the technical variation introduced during sample preparation, and to be able to obtain high-coverage transcriptomes. For bioinformatics tools specific for single-cell analysis, we refer to [128].

## Concluding remarks

Gene expression of coding RNA molecules is a complex process regulated not only at transcriptional and post-transcriptional level, but also during and after translation. To fully characterize this process on a genome-wide scale and at a nucleotide level, numerous high-throughput RNA profiling sequencing methods have been developed. The determination of the actual structure of a transcript cannot be achieved without capturing different processing and regulatory events occurring in the same transcript. Capturing these events by combining different complementary methods comes with limitations, due to the uncertainty faced while trying to reconstruct the transcript. Technological advances in the sequencing field are leading to full-length transcript sequencing. From a technological point of view, it is already possible to sequence full-length cDNA molecules, even though future improvements in the production of cDNA molecules are still required to fully investigate the exact structure of each transcript variant. Full-length transcript sequencing will help defining any coupling between the different layers of regulation of gene expression and lead to a better understanding of the complexity of the transcriptome and its expression. Direct use of RNA as a template for sequencing will further reduce biases introduced in the sample preparation procedure. The final outcome of gene expression could not be fully characterized without information on the translatome. Ribosome profiling represents the newest, most exciting tool to study gene expression at the level of translation. The use of a combination of approaches focusing at transcriptional, post-transcriptional and translational level will help to comprehensively characterize gene expression regulation.
